# Evaluation of push-out bond strength, dentinal tubule penetration and adhesive pattern of bio-ceramic and epoxy resin-based root canal sealers

**DOI:** 10.1371/journal.pone.0294076

**Published:** 2023-11-13

**Authors:** Mohmed Isaqali Karobari, Rumesa Batul, Niher Tabassum Siddiqua Snigdha, Matheel AL-Rawas, Tahir Yusuf Noorani

**Affiliations:** 1 Department of Restorative Dentistry & Endodontics, Faculty of Dentistry, University of Puthisastra, Phnom Penh, Cambodia; 2 Dental Research Unit, Center for Global health Research, Saveetha Institute of Medical and Technical Sciences, Saveetha University, Chennai, Tamil Nadu, India; 3 Conservative Dentistry Unit, School of Dental Sciences, Universiti Sains Malaysia, Health Campus, Kubang Kerian, Kota Bharu, Kelantan, Malaysia; 4 Department of Paediatric Dentistry, School of Dental Sciences, Universiti Sains Malaysia, Health Campus, Kubang Kerian, Kota Bharu, Kelantan, Malaysia; 5 Prosthodontics Unit, School of Dental Sciences, Health Campus, Universiti Sains Malaysia, Kubang Kerian, Kota Bharu, Kelantan, Malaysia; Universidade Federal Fluminense, BRAZIL

## Abstract

**Introduction:**

Root canal sealing materials play a crucial role in an endodontic procedure by forming a bond between the dentinal walls and the gutta-percha. The current study aims to analyse the dentinal tubule penetration and adhesive pattern, including the push-out bond strength of six commercially available root canal sealers.

**Methodology:**

Eighty-four mandibular first premolars were split into seven groups (and n = 12), Group 1: Dia-Root, Group 2: One-Fil, Group 3: BioRoot RCS, Group 4: AH Plus, Group 5: CeraSeal, Group 6: iRoot SP, Group 7: GP without sealer (control). Two groups were made, one for dentinal tubule penetration and the other for push-out bond strength; the total sample size was one hundred sixty-eight. Root canal treatment was performed using a method called the crown down technique, and for obturation, the single cone technique was used. A confocal laser scanning microscope (Leica, Microsystem Heidel GmbH, Version 2.00 build 0585, Germany) was used to evaluate dentinal tubule penetration, and Universal Testing Machine was utilised to measure the push-out bond strength (Shimadzu, Japan) using a plunger size of 0.4 mm and speed of 1mm/min. Finally, the adhesive pattern of the sealers was analysed by HIROX digital microscope (KH-7700). Statistical analysis was carried out by a one-way Anova test, Dunnet’s T3 test, and Chi-square test.

**Results:**

Highest dentinal tubule penetration was noticed with One-Fil (p<0.05), followed by iRoot SP, CeraSeal, AH Plus, Dia-Root also, the most negligible value was recorded for BioRoot RCS. Meanwhile, BioRoot RCS (p<0.05) demonstrated the greater value of mean push-out bond strength, followed by One-fil, iRoot SP, CeraSeal, AH Plus and Dia-Root. Regarding adhesive pattern, most of the samples were classified as type 3 and type 4 which implies greater sealing ability and better adherence to the dentinal wall. However, BioRoot RCS revealed the most type 4 (p<0.05), followed by AH Plus, One-Fil, CeraSeal and Dia-Root.

**Conclusion:**

The highest dentinal tubule penetration was shown by One-Fil compared to other groups. Meanwhile, BioRoot RCS had greater push-out bond strength and more adhesive pattern than other tested materials.

## 1. Introduction

Fundamental to a successful endodontic treatment procedure is the complete debridement of the contaminated root canal system, shaping and tri-dimensional canal space sealing to avoid reinfection of the pulpal cavity. Improper sealing of any prepared canal space and inadequate removal of microorganisms resulting in the persistence of infection in the canal or the peri-radicular region are the two principal reasons for the failure of root canal treatment [1, 2]. Gutta-percha is regarded as the “gold standard” for obturation but does not seal the canal space entirely because it does not bond to the dentinal wall resulting in the leakage of the prepared root canal [3]. Thus, an endodontic sealer is essential in establishing a tight bond between the dentinal wall and the core material [4]. Over the past decade, various endodontic sealers have been introduced in dentistry, like calcium hydroxide-based, zinc oxide eugenol-based, silicone and methacrylate-based, glass ionomer cement-based, resin-based, MTA-based including bio-ceramic calcium silicate-based sealers [5].

Bio ceramics are biocompatible, inorganic, and non-metallic material consist of zirconia, alumina, hydroxyapatite, glass ceramics and calcium phosphate. Krell and Wefel were the first to use bio ceramic as the root sealer efficiently [6]. They can resorb or regenerate the tissues along with greater sealing ability and antibacterial property [7]. Bio ceramic root canal sealers contains calcium phosphate which improves their setting properties and forms a crystalline structure which is chemically similar to tooth thereby enhancing the bonding of root dentin to sealer and improving the bond strength [8].

iRoot SP (Innovative BioCeramix, North Fraser Way Burnaby, Canada), Dia-Root (DiaDent, Chungcheongbuk-do, Korea), and CeraSeal (Meta Biomed, Korea) are calcium silicate-based bioceramic sealers that chemically bond to dentine and establish a bond among obturating material together with dentin walls to create a hermetic seal. These aluminium-free sealers have good physico-mechanical properties such as biocompatibility, radiopacity, hydrophilicity, and dimensional stability. Moreover, they do not expand or shrink with greater push-out bond strength [9, 10]. BioRoot RCS (Septodont, Saint-Maur-des-Fossés Cedex, France), another bioceramic sealer, was introduced in 2016. It has similar properties as mentioned before [11]; however, push-out bond strength was higher than other materials [5, 12, 13]. Another recently introduced bioceramic sealer is One-fil (Mediclus, Chungbuk, Korea.), whose push-out bond strength test has not yet been reported to the best of our knowledge. AH Plus (Densply, Konstanz Germany), an epoxy resin-based sealer, is well-investigated and regarded as the standard for comparing different materials [14]. Its Physical, biological, and antimicrobial properties are favourable. Furthermore, it is reported to have higher chemical bonding and push-out bond strength [14, 15].

Dentine tubule penetration measures the sealer’s capability to penetrate dentinal tubules, an important feature required to improve the bond between sealer and dentine [16]. It is performed using a Confocal laser scanning microscope where the sealer’s penetration is recorded, added to a given circumference, and divided by the total circumference of the canal [17]. Push-out bond strength is retardation to the dispossession of filling material to the dentinal wall of the root. It is a mechanical test that records the interfacial bonding strength of sealer material to root dentin [2, 18]. The procedure involves the application of tensile load longitudinally to the root section until the gutta-percha and sealer are dislodged. It does not require sophisticated equipment as it can be performed within the root canal, which makes it superior to other tests such as shear and tensile bond strength [19].

The primary aim of a root canal sealer is to establish a bond between root dentine and gutta-percha. This bond ensures the long-time success rate of the treatment [20]. Bonding between material and tooth surface is mainly affected by the physical properties and chemical composition of sealer material, temperature and moisture control [21]. Hence, when a sealer’s bonding to root dentin is well established, it will increase push-out bond strength and display favourable adhesive patterns [12].

Several investigations have been carried out to establish the push-out bond strength of endodontic sealers with varying results [22–25], but the knowledge of dentine tubule penetration is limited. Furthermore, to the best of our knowledge, no other study has been organised on the adhesive pattern of different bioceramic sealers tested in this study. Hence, the present in-vitro study aimed to determine the dentinal tubule penetration, push-out bond strength and evaluate the adhesive pattern of teeth obturated with different bioceramic based root canal sealers.

## 2. Materials and methods

The study was conducted in accordance with the Declaration of Helsinki and exempted by the Institutional Review Board Human Research Ethics Committee of Universiti Sains Malaysia, Universiti Sains Malaysia, with a number (USM/JEPeM/21060495). Informed consent was waived by the ethics committee, due to non-interventional nature of the current study. Additionally, the patients sign a general consent before any investigation or treatment is rendered, including consent to use the extracted teeth in future studies without any personal identification of patients. Bivariate normal distribution and correlation were used to determine the sample size (G*Power 3.1.9 for Windows). A minimum of 12 teeth in each sealer group was determined. In each group, 15% of the samples were added for potential drop-out, and the ultimate sample number was 84 for each test group. The samples were split into two separate groups considering the test performed. One group was for Confocal Lesser Scanning Microscopy (Group A), and another one was for Push-out Bond Strength Test (Group B) [12]. Hence, the total sample size was 168. The teeth included were freshly extracted mandibular first premolars from patients aged 20–40.

### 2.1 Sample selection

All the teeth were scrutinised at 20x magnification under the microscope (Leica Microsystem Imaging Solution, Cambridge) to confirm they were free from crown and root caries, fracture, restoration, abrasion, and root resorption. A digital radiograph was taken to ensure a developed apical foramen, a single canal, and Vertucci’s Type 1 classification. The digital radiographic software (Planmeca Romexism 2.9.2R, 2012) was used to measure the tooth length from the digital radiograph. Teeth with an overall length of 21mm (± 1mm) (tip of the buccal cusp to apical foramen) as well as a root length of 12mm (± 1mm) were included [26]. All the selected samples were cleaned using an ultrasonic scaler to remove calculus and stain. To eliminate leftover tissue debris, teeth were submerged in a 2.5% sodium hypochlorite (NaOCl) solution for 24 hours (Lenntech, Delfgauw, Netherlands).

### 2.2 Endodontic preparations

Access cavities of 168 teeth were performed using an Endo-Access bur (Dentsply Maillefer, Switzerland), number 4. 25mm length 10 k-file (FlexOFiles; Dentsply Maillefer, Switzerland) has been utilised to ensure the patency of canal and working length were confined with the help of apical foramen. The root canal was prepared with a rotary machine (Dentsply, x-smart, Japan) and a single rotary file system (XP-endo shaper, Switzerland) to a final root canal apical size of size 30 and 4% taper. Spontaneous irrigation was done to remove all the debris during instrumentation using 2.5% NaOCl and first and last irrigation with 17% EDTA (ethylenediaminetetraacetic acid) (Promega Corporation, Wisconsin, USA) solution to eliminate the smear layer. The teeth were irrigated using normal saline (10 ml) (RMBIO, Missoula, Montana) to flush out the remnant chemical irrigation solution. Canals were dried with 30-sized paper points (Dentsply, Maillefer, USA). The samples were allocated into two test groups using a random allocation method. Each test group was divided into seven sealer groups with 12 samples each and obturated using one of the preselected root canal sealers listed below. The particulars of the root canal sealers employed in the present study are recorded in Table 1.

**Table 1:** Characteristics of root canal sealers of the current study.

Material	Manufacturer	Composition	Batch number
Dia-Root	DiaDent, Chungcheongbuk-do, Korea.	Calcium silicate, Ytterbium trifluoride, Calcium aluminate, Silanamine, 1,1,1-trimethyl-N-(trimethylsilyl)-, Zirconium dioxide, hydrolysis products with silica, Polyethylene glycol 400, Polyethylene glycol 200 along with Light mineral oil, Hydroxypropyl methylcellulose, Polyoxyethylene (20) sorbitan monooleic acid.	BS2111171
One-Fil	Mediclus, Chungbuk, Korea.	Calcium silicate, H_2_O	OS12T1432
BioRoot RCS	Septodont, Saint-Maur-des-Fossés Cedex, France	Tricalcium silicate, excipients in powder form, zirconium oxide (opacifier), and calcium chloride, as well as excipients as an aqueous liquid	B27972
AH Plus	Densply, Konstanz Germany.	Epoxide paste: calcium tungstate, diepoxide, aerosil, zirconium oxide, including pigment. Amine paste: zirconium oxide, aerosil, 1-adamantane amine, calcium tungstate, N, N0 -dibenzyl-5-oxa-nonandiamine-1,9, TCD-diamine along with silicon oil	2105000051
CeraSeal	Meta Biomed, Korea	Zirconium oxide, Calcium silicate, including Thickening agent	CSL2112281
iRoot SP	Innovative BioCeramix, North Fraser Way Burnaby, Canada.	Calcium hydroxide, calcium phosphate monobasic, zirconium oxide, including filler agents	21004SP

Group 1 (n = 12)–Gutta-percha (GP) and Dia-Root

Group 2 (n = 12)–GP and One-Fil

Group 3 (n = 12)–GP and BioRoot RCS

Group 4 (n = 12)–GP and AH plus

Group 5 (n = 12)–GP and CeraSeal

Group 6 (n = 12)–GP and iRoot SP

Group 7 (n = 12)–GP only (control group)

The GP cone size 30, 4% tapered (Dia Dent, Dia Dent group International, Korea), was tested in the root canal to confirm the apical ’tug-back’ ahead of obturation. Obturation was performed by a single cone technique. The weight of each sealer was recorded by a digital scale, with the proportion of dye being 1% of the sealer weight. The sealers were manipulated following the guidelines provided by the manufacturer and mixed with 1% of rhodamine B dye (Sigma Aldrich Co., St Louis, MO, USA) for the Confocal Lesser Scanning Microscopy group (Group A). The obturation was carried out without the dye for the push bond strength group (Group B). A sealer layer was applied to the canal wall using the master cone and planted inside the respective canal. After that, a heated System B plugger (Xtra Fine .4 size) was used to severe and seal the coronal portion of the GP.

After obturation, the access cavity wall was etched using 37% orthophosphoric acid (Kerr Corporation, Orange, CA), for 15s, followed by 30s of wash. Next, a bonding agent was applied to the cavity wall. Finally, curing was performed using a light cure machine for 10s. Subsequently, cavities were restored with micro-hybrid resin composite (Zhermack, universal composite, Zhermack S.p.A, Italy) by applying a 2mm composite layer followed by light curing for 40s. A composite polishing kit was used to polish the final restoration. A single expert operator carried out all the sample preparation to redact the inner bias of the current study. Every sample was place in the incubator (by Memmert GmbH +Co. KG, Schwabach, Germany) for 72 hours at 37-degree Celsius temperature and 100% humidity to allow complete setting of all the sealers.

After incubation, the teeth were sectioned horizontally by a hard tissue cutter (EXAKT 312, EXAKT Technologies, Inc., Oklahoma City, USA). Slices were obtained from within 5 mm to 7 mm from the root apex, considered the middle 3^rd^ of root canals [12]. The width of the slices was around 1±0.1 mm. All the samples in Group A and B were examined under the microscope to ensure the slices were not cracked and the sealer was void-free.

### 2.3 Confocal laser scanning microscopy

One slice from each sample was selected from sealer groups of group A for confocal laser scanning microscopy (Leica, Microsystem Heidel GmbH, Version 2.00 build 0585, Germany). The selected sample slice was placed on a slide under the microscope, and 10x magnification was used to capture the image. Penetration of sealer in the dentinal tubule was measured in each sample using Leica (Microsystem Heidel GmbH, Version 2.00 build 0585, Germany) software to measure the most extended penetration depth of sealer in each sample [12]. After the measurement, an average value was obtained for each sample.

### 2.4 Push-out bond strength test

One slice of each sample was selected from sealer groups of group B for performing push-out bond strength test using a Universal Testing Machine (manufactured by Shimadzu, Japan) until the gutta-percha material was displaced. A compressive force was used on each obturated sample slice by a cylindrical stainless-steel plunger of 0.4 mm in the apico-coronal direction. The speed of the machine was selected as 1mm per minute. The force applied to the material to dislocate the GP was measured in Newton (N). The formula of push-out bond strength calculation is the push-out strength [15] is equal to the Maximum applied force (N) by the surface of root dentine walls (mm2). The surface area (mm^2^) of each section was calculated with the help of the conical frustum surface area formula:

$$\pi \text{x}\;\left(\text{r}1+\text{r}2\right)\;\surd \left(\text{r}1-\text{r}2\right)2+\text{h}2$$

In this context, r1 represents the radius at the coronal portion, r2 represents the radius at the apical portion, and h denotes the width of the sample, which is measured to be 1±0.1 mm.

### 2.5 Assessment of adhesive pattern

This assessment used the same sample slice for push-out bond strength. The remaining sealer on the dentinal wall was analysed under a HIROX digital microscope (KH-7700) to confirm the adhesive pattern. The images were captured at 100x and 200x magnification. The adhesive pattern of sealer was classified into four types based on the classification introduced by Lin et al. [5] in 2021.

Type 1: sealer is noted in one quadrant.

Type 2: sealer is noted in two quadrants.

Type 3: sealer is noted in three quadrants.

Type 4: sealer is noted in all four quadrants.

If no sealer is present on dentinal walls, that will be considered ’non-adhesive’ [5].

### 2.6 Statistical analysis

Statistical analysis was performed by SPSS version 26.0 for Windows (SPSS Inc., Chicago, IL, USA). The normality test to determine data analysis was done by the Kolmogorov-Smirnov test. As data distribution was normal, the data of push-out bond strength along with dentinal tubule penetration were analysed by One Way ANOVA, including Post Hoc Analysis with Dunnett’s T3 test. A chi-square test assessed the differences in adhesive and cohesive failure among all the endodontic sealers. The researchers established the significance level of p = 0.05.

## 3. Results

### 3.1 Qualitative analysis of dentinal tubule penetration

**Fig 1:**
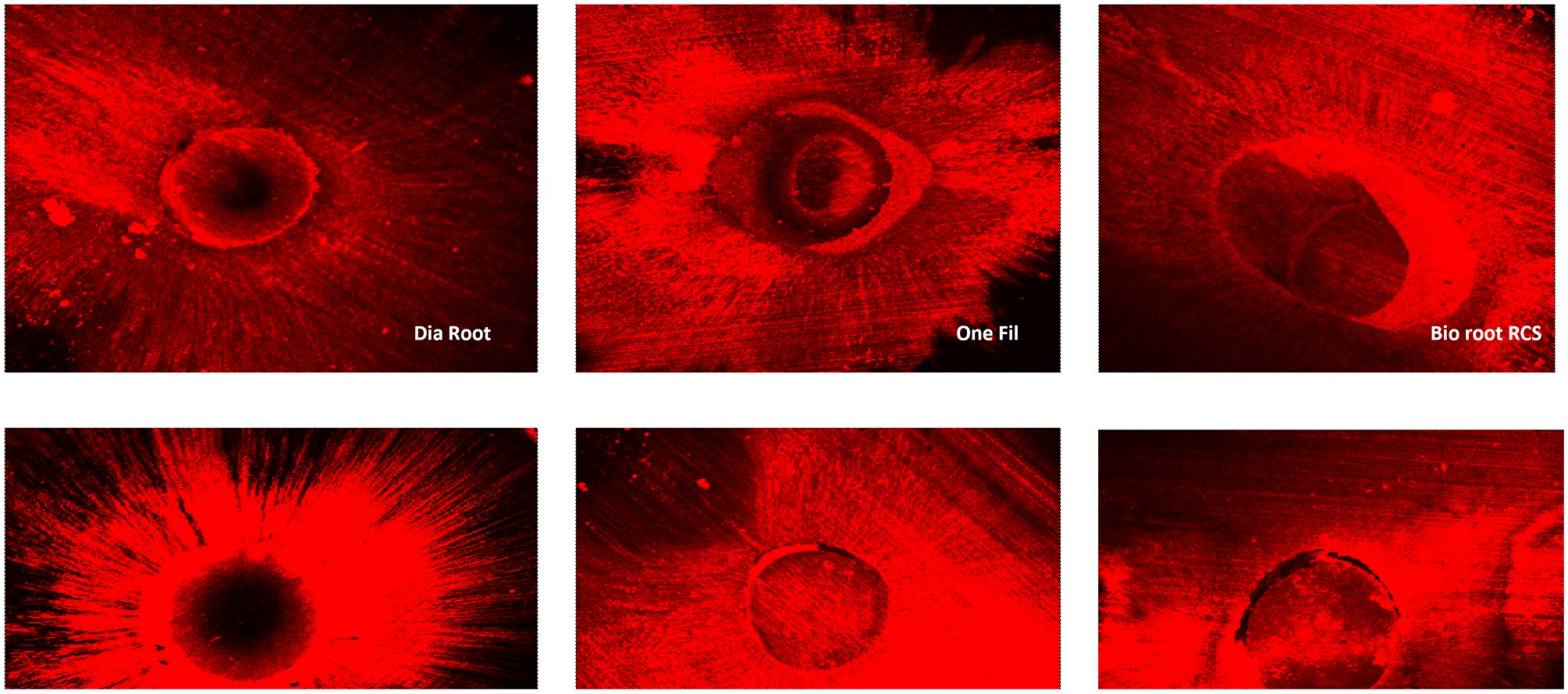
Confocal laser scanning microscope images of root canal sealers from the middle third of the root.

Confocal Laser Scanning Microscope was utilised to assess dentinal tubule penetration of root canal sealers. The results (p<0.05) of all the sealers are tabulated in Table 2 (Fig 1). The most extended dentinal tubule penetration was demonstrated by One-fil, followed by iRoot SP, CeraSeal, AH Plus, Dia-Root and the most negligible value was recorded by BioRoot RCS.

**Table 2:** Dentinal tubule penetration of the test groups.

Material	N	Mean	Median	Std. Deviation	Minimum	Maximum	p-value
Día-Root	12	287.5279	295.7855	49.26845	206.02	369.33	0.008*
One-Fil	12	339.9795	344.5340	58.36894	268.42	469.67	
BioRootRCS	12	231.7183	224.1095	66.20950	119.55	346.99	
AH Plus	12	289.1567	272.4845	115.72966	147.49	567.13	
CeraSeal	12	322.6750	300.6700	82.35174	157.84	425.28	
iRoot SP	12	334.3773	330.6775	61.12119	200.17	467.98	
Total	72	300.9058	299.9740	81.71728	119.55	567.13	

*Significant p-value <0.05

The analysis of inter-group comparison yielded a statistically significant disparity between BioRoot RCS-One fil and Bio-Root-iRoot groups with p<0.05; meanwhile, all other groups demonstrated no statistically prominent difference with p>0.05 (Table 3).

**Table 3:** Intergroup comparison of sealers with dentinal tubule penetration.

Material	Std Error	95% Confidence Interval		p-value
		Lower Bound	Upper Bound	
BioRootRCS-One-Fil	25.47978	25.3638	191.1586	0.005*
BioRootRCS- iRoot SP	26.01201	-187.2060	-18.1120	0.010*

*Significant value <0.05; Anova One Way; Post Hoc Analysis with Dunnett’s T3 test show all pair comparison is >0.05 except Bio Root-One fil and Bio Root-I root <0.05.

### 3.2 Evaluation of push-out bond strength

The results of push-out bond strength are tabulated in Table 4. A significant difference in the push-out bond strength is noted in the sealers (p<0.05) and Bio root denoting the greater mean push-out bond strength value followed by One-Fil, iRoot, CeraSeal, AH Plus and Dia-Root (Table 4). The inter-group comparison revealed a statistically significant difference between Día-Root-Onefil, Día-Root-BioRoot RCS, Día-Root-CeraSeal and Día-Root-iRoot with p<0.05; meanwhile, all other groups demonstrated no statistically compelling difference with p>0.05 (Table 5).

**Table 4:** Push-out bond strength of all test groups.

Material	N	Mean	Median	Std. Deviation	Minimum	Maximum	p-value
Día-Root	12	0.3644	0.3510	0.23693	0.09	0.77	<0.001*
One-Fil	12	0.9798	0.8530	0.43176	0.48	1.88	
BioRootRCS	12	1.1806	1.1465	0.44262	0.63	2.18	
AH Plus	12	0.6085	0.6423	0.15562	0.32	0.78	
CeraSeal	12	0.6734	0.6285	0.17135	0.44	0.94	
iRoot SP	12	0.7537	0.7610	0.21256	0.43	1.22	
Total	72	0.7601	0.7035	0.39103	0.09	2.18	

*Significant p-value <0.05

**Table 5:** Intergroup comparison of sealers with push-out bond strength.

Material	Std Error	95% Confidence Interval		p-value
		Lower Bound	Upper Bound	
DiaRoot-One-Fil	0.14217	-1.0912	-0.1396	0.006*
DiaRoot- BioRoot RCS	0.14493	-1.3022	-0.3302	0.000*
DiaRoot-Ceraseal	0.08441	-0.5860	-0.0321	0.022*
DiaRoot- IRoot SP	0.9819	-0.6881	-0.0905	0.005*

*Significant value <0.05; Anova One Way; Post Hoc Analysis with Dunnett’s T3 test show all pair comparison is <0.05 for Día Root-One-Fill, Día Root-Bio Root, Día Root-Cera Seal and Día Root-I Root, all other pairs >0.05.

### 3.3 Assessment of adhesive pattern

Table 6 demonstrates the adhesive pattern of all sealer groups. According to the classification, type 4 indicates the most favourable type for sealer material (p<0.05). Most samples fell in type 3 and type 4. However, BioRoot RCS demonstrated the most type 4 followed by AH plus, One-Fil, CeraSeal and Dia-Root (Table 6). The inter-group comparison revealed a statistically compelling variation between Día-Root-One-Fil, Dia-Root-BioRoot RCS, and Dia-Root-AH Plus with p<0.05; meanwhile, all other groups reported no significant variation with p>0.05 (Table 7). The data of control groups is not added to any table because samples of the control group were only sealed with GP without sealer. There was no penetration of sealer, the result of push-out bond strength was 0, and no sealer residue was noted in the adhesive pattern.

**Table 6:** Pattern of adhesiveness of all tested endodontic sealers.

Type of Sealer	Type of Adhesive Pattern					p-value
	Non-Adhesive	Adhesive				
		Type I	Type II	Type III	Type IV	
Dia-Root	1	5	2	3	1	0.001*
One-Fil	0	0	0	8	4	
BioRoot RSC	0	0	0	3	9	
AH Plus	0	0	2	3	7	
CeraSeal	1	1	1	6	3	
iRoot SP	2	1	1	1	7	
Total	4	7	6	24	31	

*Significant value <0.05

**Table 7:** Intergroup comparison of sealers with adhesive pattern.

Material	Std Error	95% Confidence Interval		p-value
		Lower Bound	Upper Bound	
Dia-Root-One-Fil	0.37268	-2.7752	-0.2248	0.016*
Dia-Root- BioRootRCS	0.36842	-3.1848	-0.6485	0.002*
Dia-Root-AH Plus	0.41363	-2.9479	-0.2187	0.016*

## 4. Discussion

Root canal sealers effectively obtain the tight seal by forming a bond between gutta-percha and the dentinal wall. They are capable of filling voids, areas that are difficult to approach, accessory canals and act as a lubricant [27].

The sealers used in our study are resin-based and bio-ceramic-based (Table 1). AH-Plus is an epoxy resin-based sealer (manufactured by Dentsply DeTrey, Konstanz, Germany) which is commonly used as comparison material for research and is regarded as the ’gold standard in endodontics due to its biological, mechanical and physico-chemical feature of being insoluble, radiopaque [14] and antimicrobial [28]. Its bond strength is higher due to micro-mechanical locking caused by covalent bonds, while polymerisation shrinkage is less [29]. Bio-sealers are easily acceptable by the tissues and form a bond with dentin as they are identical to bone and teeth, concerning their chemical composition and structure [30]. They are hydraulic sealers containing calcium silicate and calcium phosphate, capable of inducing mineralisation and tissue repair [28, 31, 32]. They are antibacterial and go through a bioactivity process resulting in apatite nucleation, which avoids removing gutta-percha inside the canal and forming a tight contact seal [33].

The bonding of sealer to the root can be determined by several microscopic methods, such as Scanning electron microscopy, light microscopy and confocal laser scanning microscopy (CLSM) [34]. However, CLSM was used in the current study to establish the depth of dentinal tubule penetration as it offers multiple advantages over the other methods. It can provide necessary information about sealer penetration within the root canal system along the dentinal tubule and allows the visualisation of depth and control of the depth by reducing or incising the background information from the focal plane, and is capable of assembling multiple optical sections despite of a thick section of specimen [34, 35]. To evaluate dentinal tubule penetration, samples need to contain fluorescent dye. In this study, rhodamine B dye (Sigma Aldrich Co., St Louis, MO, USA) acts as an indicator for visualisation under a confocal laser scanning microscope, and it is non-reactive with the chemical and physical properties of all sealers in this study [34].

In the current study, One-Fil expressed greater dentinal tubule penetration, and BioRoot RCS had the least penetration (Table 2). The further inter-group comparison reported a statistically prominent difference between BioRoot-One fil and Bio-Root-iRoot groups. Meanwhile, all other groups had no significant difference (Table 3). Türker S.A. et al. (2018) used BioRoot RCS, AH 26 and MTA plus as root canal sealers, whereas BioRoot RCS showed less penetration because it has lower fluidity when it comes in contact with dentinal tubule compared to other materials used in the study [23]. In contrast, Kim Y et al. [36] recorded higher dentinal tubule penetration of BioRoot RCS compared to other tested materials in the coronal and middle third; Khullar et al. also noted similar results [37]. OneFil displayed the highest penetration among all the tested materials in the present study. To the best of our knowledge, no study has been performed on the dentinal penetration of OneFil sealer in the literature. However, the highest reading can be attributed to the penetration property of sealer (calcium silicate-based) inside the dentinal tubules without using extra intra-canal pressure for compaction, which is generally not applied in the single-cone technique [38]. AH Plus exhibited less penetration of sealer inside the dentinal tubules than other tested calcium silicate-based bioceramic sealers. This result follows the other studies, which stated that smaller particles of calcium silicate types of cement and their uniform occupation in the intra-tubular space helped better penetration [17, 29, 38, 39]. In another study, AH Plus had the deepest tubular penetration in the apical third compared to other sealers due to the continuous wave technique [36]. Hence, penetration of sealers is affected by the size of the particles, the material’s solubility and viscosity [40]. Further obturation technique and canal anatomy of teeth are other essential factors to consider [38]. The present study included only mandibular first premolars, another element to make variations; hence further studies should be conducted on root canals which have different anatomy to ensure the results.

Apart from the properties mentioned above, push-out bond strength is another critical feature of sealers. However, it is a variable used to analyse the bonding of sealers with core material and dentinal wall [41]. Push-out test is used to analyse the bond strength of different materials; however, it ultimately does not reflect the performance of the sealer as there are various factors responsible for the fluctuation of the result and affecting the mechanical bond strength of the sealer directly, such as canal diameter, applied load, punch diameter and position of specimen [20, 42]. However, precise machining with a steady diameter and filling the sealers in canals can benefit the result [42]. Besides, a single tooth sample can be used for assessing the push out bond strength of root canal sealers [43]. However, obturating the teeth with sealer and gutta percha as carried out in the current study would help in mimicking the clinical scenario in an appropriate manner.

Universal Testing Machine (Shimadzu, Japan) was used in the current study to analyse the push-out bond strength of the sealers (Table 1) with a cylindrical stainless-steel plunger of 0.4 mm, and the speed of the machine was 1mm per minute. Among the tested sealers BioRoot RCS had the greater push-out bond strength result, while CeraSeal displayed the least value (Table 4). Inter-group comparison displayed statistically significant differences between Dia Root-OneFil, Dia-Root-BioRoot, Dia-Root-CeraSeal and DiaRoot-IRoot (Table 5). The highest value of BioRoot RCS can be attributed to the nature of calcium silicate cement, capable of forming a ’mineral infiltration zone’, and hydration products of calcium silicate can cause degradation of interfacial dentin, which forms a porous structure allowing higher penetration of calcium and hydroxyl ions. This micromechanical interaction is mainly responsible for the stronger adhesion of BioRoot RCS compared to other sealers[5, 41, 44, 45]. These results are consistent with other studies [5, 20, 22, 23].

Although CeraSeal has the constructive features of bio-ceramic sealers [46], it denoted the least value, this could be due to the difference in the composition of the sealers affecting the bonding of CeraSeal [2]. Despite the presence of calcium silicate as the main component in all the tested sealers, the difference in its percentage or presence of other constituents may have affected the results. Furthermore, different methodologies could also cause value differences among the current study as well as other studies [10, 46, 47]. AH Plus denoted lesser push-out bond strength than BioRoot RCS along with most other calcium silicate-based sealers (Table 4). This outcome follows other studies [22]. Epoxy resin-based sealers have lesser adhesion to the root than calcium silicate sealers [23], and deficient adherence between the sealer and gutta-percha can cause leakage, further leading to fluid penetration at the interface [48]. In contrast to this, the higher push-out bond strength of AH Plus was revealed as to other calcium silicate sealers [15, 41]. The explanation for this variation could be using different irrigation solutions that compromise the bonding of calcium silicate types of cement; further factors like slice thickness and load velocity can affect the results [15, 22].

Another objective of the present study was to assess sealers’ adhesive pattern, which implies their bonding ability [19]. No previous studies were conducted to assess the adhesive pattern of different sealers; hence this is a novel work which aims to determine the sealer in four divided quadrants using HIROX digital microscope (KH-7700). If the sealer is present in one quadrant, it will be under type 1; if found in two, it will be noted as type 2 and followed by a similar pattern. Sealer found in all four quadrants implies a good adhesive nature, while no sealer indicates better cohesiveness [5]. In the present study, BioRoot RCS had most type 4 samples (Table 6) following its bonding ability with dentine, as explained earlier [10, 24, 41].

On the other hand, Dia-Root displayed the poorest adhesive patterns. The inter-group difference revealed a statistically prominent variation between Dia-Root-Onefil, Dia-Root-BioRoot, and Dia-Root-AH Plus (Table 7). The higher adhesiveness of BioRoot RCS can be attributed to its superior push-out bond strength value to root dentin (Table 3). Adhesion of sealer is affected by several factors like the surface tension of adhesive, i.e., root sealer, surface energy and clean surface of adherend which can be root dentin or gutta percha and the capacity of adhesive to moisten the surface [12, 49].

This research does have certain limitations. Since this was an in vitro study, the findings may not be generalisable to clinical dental situations. Six different commercial sealer brands were used in the experiment. It is possible that other brands would not yield the same results. Future research must examine brands other than those employed in our investigation to set a baseline for fair comparisons. Additionally, NaOCl used for disinfection of the teeth samples may have affected the results of this study [50]. However, it is worth noting that, NaOCl was not directly in contact with the root dentine surface since the whole teeth were immersed in NaOCl. Besides, the push out force was applied to the gutta percha and sealer and not to the dentine. Thus, the strength of dentine was not tested directly. Despite that, the possible effect of NaOCl on the root dentine, would be standardised to all samples having little possible effects on results of the study. During the sectioning of the roots, it is likely that some of the dye spread to the external root surface during handling. However, it can be noted that the sealer penetration was not disturbed as the continuity between the florescence on the external surface and the dye penetrating in the dentinal tubules cannot be seen in any of the sample. More research is necessary to assess the unique experimental sealer’s long-term adhesion, penetration and sealing ability in different levels of the root canal, bond strength, incorporation of fluorescence dyes, clinical performance, and physical and chemical properties. Nevertheless, the findings of this study provide some clinical relevance on the choice of commercially available bioceramic based root canal sealers on the basis of their ability to bond and penetrate deeper into the dentinal tubules to provide an adequate seal.

## 5. Conclusions

Within the study’s limitations, the greater push-out bond strength and the best adhesive pattern were shown by BioRoot RCS, a bioceramic sealer, compared to other tested materials. These results are attributed to the property of bio-ceramic sealer capable of forming micromechanical bonds with the root dentin and providing better adhesion. On the other hand, One-Fil exhibited the highest dentinal tubule penetration compared to other groups. AH Plus (epoxy resin-based sealer) used in the present study revealed greater dentinal tubule penetration than BioRoot RCS but lesser than other calcium silicate sealers. However, the push-out bond strength and adhesive patterns were lesser than BioRoot RCS.

## Supporting information

S1 Data (XLSX)
