# Correction: Evaluation of push-out bond strength, dentinal tubule penetration and adhesive pattern of bio-ceramic and epoxy resin-based root canal sealers

**DOI:** 10.1371/journal.pone.0295461

**Published:** 2023-11-30

**Authors:** Mohmed Isaqali Karobari, Rumesa Batul, Niher Tabassum Siddiqua Snigdha, Matheel AL-Rawas, Tahir Yusuf Noorani

The affiliation for the fifth author is incorrect. Tahir Yusuf Noorani is not affiliated with #2 but with #3: Conservative Dentistry Unit, School of Dental Sciences, Universiti Sains Malaysia, Health Campus, Kubang Kerian, Kota Bharu 16150, Kelantan, Malaysia.
